# Career identity and career success among Chinese male nurses: The mediating role of work engagement

**DOI:** 10.1111/jonm.13782

**Published:** 2022-09-22

**Authors:** Chao Wu, Mi‐mi Fu, Si‐zhe Cheng, Ya‐wei Lin, Jia‐ran Yan, Jing Wu, Xin‐yan Zhang, Bao‐hua Cao, Juan Du, Hong‐juan Lang

**Affiliations:** ^1^ Department of Nursing Fourth Military Medical University Xi'an China; ^2^ Department of Pharmacy Sanya Rehabilitation Center Sanya China; ^3^ Department of Military Medical Psychology Fourth Military Medical University Xi'an China; ^4^ Department of Engineering Army 75 Group Military Hospital Kunming China

**Keywords:** career identity, career success, male nurses, work engagement

## Abstract

**Aim:**

This study aimed to investigate the effect of career identity on career success among Chinese male nurses and to examine the mediating role of work engagement in this relationship.

**Background:**

Recently, with the development of the nursing career, male nurses take up a higher share and play a more important role in the nursing team. With its own particularity and advantages, this group's stability closely relates to the future of the nursing team. Therefore, promoting the career success of the male nurses is essential to the nursing team development.

**Methods:**

The data were collected in China. A sample of 557 male nurses completed measures of career identity, work engagement and career success scale. Structural equation model was adopted to verify the research hypotheses.

**Results:**

Career identity was significantly and positively related to male nurses' work engagement and career success (*p* < .01). And work engagement partially mediated the association between career identity and career success.

**Conclusion:**

Career identity is critical to predicting and enhancing male nurses' career success. Work engagement plays an intervening mechanism explaining how career identity promotes career success among male nurses.

**Implications for Nursing Management:**

Nursing management should minimize the impact of the traditional concept, implement the gender equality and provide moderate care for male nurses to facilitate balanced development of gender by upgrading the management system. The administrators should carry out skill training based on male nurses' features and the need of the department. Given full play to their respective advantages, male nurses will make great progress in professional development and achieve greater career identity and work engagement. Meanwhile, the further exploration of better incentive mechanism also makes sense in improving career identity and work engagement by the reform of performance appraisal mechanism and salary adjustment according to their ability.

## 1 INTRODUCTION

With the development of the medical and health services, the nursing career has made great progress in the development of quantity and quality (Lu et al., 2018) and with the development of society and nursing specialty, more and more male nurses take this position gradually. However, nurse shortage is still a big problem being experienced around the world (Beitz, 2019). Male nurses, compared with female nurses, enjoy some gender advantages with better performance in physiology and psychology (Mao et al., 2021). In recent decades, male nurses have been an irreplaceable part of the nursing team, and to some extent, their appearance alleviated the shortage of nurses (Stanley et al., 2016). According to the survey, the rate of male nurses in western countries is between 10% and 30% (Younas & Sundus, 2018). Male nurses in China, whose development is relatively late but quick, are still not in line with those in western countries in proportion with only about 5% (Stanley et al., 2016). Despite the surging demand from the society, male nurses still share the problem of low career identification, higher mental stress and lack of job planning due to the impact of traditional concept, improper self‐awareness and low income (Liu et al., 2018). The group of male nurses is not stable and has a high turnover rate, which affects the quality and safety of patient care and becomes a stumbling block to the development of nursing career (Sasa, 2019; H. Zhang & Tu, 2020).

Career success refers to the positive psychological reception accumulated in one's own career development and the relative achievements (Brownrout et al., 2021; H. Zhang & Tu, 2020). It is an important indicator evaluating individual development of professional career (Tu & Okazaki, 2021). When it turns to nurses, it refers to positive psychological experience accumulated in nursing and sense of accomplishment related to hands‐on experience (Dan et al., 2018; Sönmez et al., 2021). A survey suggests that career success of nurses plays an important role in decreasing the turnover rate and improving quality of nursing service (Wu et al., 2020; Xu et al., 2021). Current research thus has a growing interest in exploring factors affecting male nurses' career development. Researches show that emotional labour and professional empowerment are the influencing factors of male nurses' career success (Lou et al., 2010; H. Zhang & Tu, 2020). Smith et al. (2021) found that gender stereotyping, prejudice and discrimination reduced career success for male nurses. Further research found that in the student stage, male nursing students' career choice confidence is significantly lower than that of female nursing students, which leads to their low sense of career success in the future (Twidwell et al., 2022).

Career identity refers to recognition and acceptation of professional roles given by the society. It also refers to positive response to the aim of one's career, social value and the other factors (Cruess et al., 2019; Fitzgerald, 2020). However, studies on the career identity of male nurses in China are far from enough. Existing studies imply that male nurses have lower career identity affected by traditional concept, especially the junior male nurses with less than 3 years of working experience who are reported having the lowest professional identity (Y. Chen et al., 2020). Career identity plays a key role in improving work enthusiasm and efficiency (L. Cheng, 2021). However, it is less clear whether career identity could promote career success among male nurses. So the first aim of this study was to examine whether career identity could promote career success among male nurses.

In addition, we attempted to explore the mechanisms through which career identity enhances male nurses' career success. Past studies have found that career identity is positively related to work engagement (Hirschi, 2012; Sun et al., 2022) and work engagement is positively related career success (Q. Chen et al., 2021). Accordingly, we believe that career identity, work engagement and career success are related, and work engagement may serve as an intervening mechanism between career identity and career success. Hence, the present study investigated the effect of career identity on male nurses' career success and explored the mediating role of work engagement among Chinese male nurses. As such, we attempted to shed light on the antecedents of male nurses' career success from the perspective of male nurses' career identity and explore under what mechanisms could this influence process happened. Therefore, carrying out career related research on male nurses is of great significance for stabilizing the nursing team and promoting the development of nursing in China.

## 2 LITERATURE REVIEW AND HYPOTHESES

### 2.1 Career identity and career success

Nursing career identity refers to the nursing practitioner's positive view and feeling to their career and the psychological condition determining one's positive occupational behaviour tendency (Foster, 2021; Landis et al., 2020). Nursing career identity mainly includes five core dimensions: (a) career awareness and evaluation, which is how nurses treat and cognize their career; (b) career social support, which they can perceive from the outside world to their career; (c) career social skills, which includes adjustment ability and interpersonal skills; (d) career frustration reaction, which means how they handle the difficulties; and (e) career self‐reflection, which indicates consistent in‐depth thought and summary of lessons and experience (Qi et al., 2021).

Studies reveals that good career identity can increase job satisfaction and decrease the job burnout and turnover rate (Mainous et al., 2018; T. Zhang et al., 2021). Here, we argue that career identity could promote career success among male nurses. First, good career identity is beneficial to their passion to career and work satisfaction (Kunhunny & Salmon, 2017). Second, work satisfaction and acknowledgement can mobilize the enthusiasm and efficiency in work. Finally, improved satisfaction, enthusiasm and efficiency can help them materialize their career value. Therefore, based on these arguments, our first hypothesis is:

Hypothesis 1 Career identity is positively and directly related to male nurses' career success.

### 2.2 Work engagement as a mediator

Work engagement is a positive, relatively stable and lasting emotional and cognitive state (Giménez‐Espert et al., 2020). Several studies have also documented that engaged nurses tend to have more passion, higher work quality and efficiency (Lourenção, 2018; Xiong et al., 2019; Ziapour & Kianipour, 2015). Nurses with good work engagement usually have better career satisfaction and lower work stress (Acea‐López et al., 2021; Labrague et al., 2020). Research among haemodialysis nurses shows that male nurses' work engagement score is higher than that of female nurses, because male nurses have better physical strength and work concentration than female nurses (Cao & Chen, 2019). However, existing studies lack enough investigation and further study in male nurses' work engagement. Male nurses have a low sense of identity and professional success, which may limit their further work engagement and the further development of gender advantages. Thus, it is of great importance to investigate the predictors and consequences of male nurses' work engagement.

Recently, nursing administrators and researchers have carried out the multi‐angle survey on nurses' work engagement. It shows that one's work engagement significantly correlates with career identity (Huang et al., 2019). Career identity is an intrinsic motivation factor of one's career development and a positive response to his career. Good career identity can strengthen nurses' sense of career benefit and result in more passion and commitment in the work (Sun et al., 2022).

In addition, active work engagement is conducive to obtain positive feedback at work and improve the level of work performance, so as to obtain a good sense of career success experience, that is, it helps to promote individual career success (Q. Chen et al., 2021; Hoşgör et al., 2021). At present, the survey investigating the male nurse's career success is limited. Research shows nurses' turnover rates ranged from 4.5% to 30.7% with an average of 18.2% (Wei et al., 2019). Due to the influence of Chinese traditional culture and low social acceptance and prejudice of male nurses, the turnover rate of Chinese male nurses was above 83.3%, which was much higher than that of Chinese female nurses' intention of 47.3% (Xian et al., 2020). The high turnover rate among male nurses is contributing to the job dissatisfaction, intention to leave and the shortage of nursing staff with high physical requirements, such as heavy work, which is more suitable for male nurses (Nantsupawat et al., 2017). Male nurses lack recognition, understanding and respect from patients, family members and friends. They always feel very confused about the future career development prospects, lacking a sense of belonging and security which is not conducive to their long‐term career development and career stability. Therefore, exploring the influencing factors of their career success is of great significance to stabilize and develop male nurses' team and facilitate the development of nursing career.

We thus expect that work engagement would play a mediating role in the association between career identity and male nurses' career success. Good work engagement is beneficial to their work satisfaction and enthusiasm. With better performance in work, they will get positive feedback and accomplishment to promote career success. Taken together, we propose that:

Hypothesis 2 Work engagement mediates the relationship between career identity and male nurses' career success.

## 3 METHODS

### 3.1 Participants and data collection

Participants were male nurses from 12 hospitals in Shaanxi, Shanxi, Yunnan and Hainan province of China. With the help of the hospital managers, surveys were sent to 582 male nurses from August to November 2021 using a convenient sampling method. The inclusion criteria were the male nurses who have obtained the professional qualification certificate of nurses of the People's Republic of China and officially worked in the hospital. The exclusion criteria were those who were not willing to participate or were absent during the survey. Prior to conducting the study, written and informed consent were obtained from the participants. The time of filling in the questionnaire which was sent by e‐mail was controlled within about 30 min. The aim and significance of this research was reiterated on the cover page. They were informed that they could withdraw from the study at any time for any reason. After the male nurses completed the self‐fulfilled questionnaire, the researchers immediately collected it from the network backstage. In the process of filling in the questionnaire, six nurses quit the survey and 19 questionnaires were found to be incomplete. After collecting the questionnaires, 557 questionnaires (95.70%) were determined to be valid.

### 3.2 Measures

#### 3.2.1 Career identity

Career identity was measured using the Nursing Career Identity Scale (NCIS) which was developed by scholars. The scale contains 30 items from five dimensions, including the career awareness and evaluation (nine items), career social support (six items), career social skills (six items), career frustration reaction (six items) and career self‐reflection (three items). Example item is ‘I believe those who devote themselves to their career will get rich rewards in the career’. A 5‐point Likert‐type scale was adopted, with a full score of 150. Higher scores indicated higher career identity. A score between 30 and 60 is generally considered to be a low level of career identity. The score between 61 and 90 is a relatively low level. Medium level is those who score between 91 and 120. The high level is between 121 and 150. The Cronbach's *α* was .97 and ranged between .73 and .92 for the five dimensions.

#### 3.2.2 Work engagement

Work engagement was measured using the Utrecht Work Engagement Scale (UWES) (Kulikowski, 2017). The scale contains nine items. Example items are ‘I totally immersed in my work’ and ‘I want to work when I get up every morning’. A 7‐point Likert‐type scale was adopted. Each item is scored from ‘1’ to ‘7’ from *none* to *everyday*. With the full score of 63, higher scores imply more commitment. The Cronbach's *α* coefficient of the scale was .93.

#### 3.2.3 Career success

Career success was measured using the career success scale (CSS) translated by Li et al., (2014) with good reliability and validity. The scale contains 11 items from two dimensions, including the career satisfaction (five items) and career competitiveness (six items). Example items are ‘I am satisfied with the progress I have made towards my promotion goals’ and ‘My unit believes I can create value for the organization because of my skill and experience’. A 5‐point Likert‐type scale was adopted. Each item is scored from ‘1’ to ‘5’ from *totally disagree* to *totally agree*. With the full score of 55, higher scores indicate higher career success. The Cronbach's *α* was .93 and ranged between .89 and .94 for the two dimensions.

### 3.3 Statistical analysis

First, we used the exploratory factor analysis to test the possible common method bias (Blackburn et al., 2005). We sorted out each item of the questionnaire and used SPSS 26.0 for exploratory factor analysis. In this study, we found that the first common factor interpretation rate was 31.46%, which was less than the critical standard of 40%. Then, we analysed descriptive statistics and internal consistency of each variable. Then we used the Pearson correlation coefficient to analyse the correlations among career identity, work engagement and career success. Finally, a two‐step procedure of structural equation modelling was adopted to analyse the mediating effects of work engagement between career identity and career success. Specifically, the measurement model and structural model were performed using Mplus 8.0 in two sequencing steps to examine our hypotheses. Then, we ran 2000 bootstrapping resamples to examine the mediator effect. The confidence interval is 95% confidence interval and does not contain 0, which signifies statistical significance (Motl & McAuley, 2009).

### 3.4 Ethical approval

This study was conducted under ethical guidelines described in the Helsinki Declaration (“Issue Information‐Declaration of Helsinki,” 2019). The ethics approval was not required because there was no unethical behaviour existed in the study and our study did not involve human clinical trials or animal experiments. Before the investigation, we explained the purpose to the participants, asked for their verbal consent before conducting the survey and signed the informed consent form with them. During the investigation, participants could terminate and withdraw from the investigation at any time and the questionnaire was completed anonymously.

## 4 RESULTS

### 4.1 Descriptive statistics and internal consistency of the measurement scales

With the assistance of the hospital administrators, 557 out of 582 participants completed the survey for a response rate of 95.70%. All the participants are male nurses. They had an average age of 31.06 years (*SD* = 5.23) and an average working year of 7.98 years (*SD* = 5.17).

Table 1 shows the descriptive statistics and internal consistency of the measurement scales. Male nurses' work engagement score was (48.50 ± 13.33), career identity score was (108.48 ± 21.05) and career success score was (22.34 ± 4.26) which indicate that Chinese male nurses have a high level of work engagement, a medium level of career identity (the score between 91 and 120 is a medium level) and a low level of career success. Their career success scores are the lowest, which will be the most important point we need to pay attention to in the following research. Our study will help Chinese male nurses achieve career success by finding out the influencing factors and action paths of their career success.

**TABLE 1:** Descriptive statistics and internal consistency of all measures used in the current study

		Mean	*SD*	Min.	Max.	Cronbach's *α*	Items
1	Work engagement	48.50	13.33	10	63	.93	9
2	Career awareness and evaluation	18.15	4.99	5	25	.92	9
3	Career social support	19.40	5.38	6	30	.87	6
4	Career social skills	37.55	9.64	11	55	.91	6
5	Career frustration reaction	32.58	6.86	10	45	.88	6
6	Career self‐reflection	21.27	4.60	7	30	.73	3
7	Career identity	22.34	4.26	6	30	.97	30
8	Career satisfaction	21.79	4.28	6	30	.94	5
9	Career competence	10.51	2.39	3	15	.89	6
10	Career success	108.48	21.05	35	150	.93	11

### 4.2 Correlational analysis of variables

Table 2 shows the Pearson correlation coefficients among male nurses' work engagement, career identity and career success in the survey. The results showed that career identity and its 5 dimensions have significantly positive correlations with career success (*r* = .83, *p* < .01; *r* = .80, *p* < .01; *r* = .82, *p* < .01; *r* = .78, *p* < .01; *r* = .76, *p* < .01; *r* = .72, *p* < .01) and work engagement (*r* = .70, *p* < .01; *r* = .68, *p* < .01; *r* = .67, *p* < .01; *r* = .68, *p* < .01; *r* = .62, *p* < .01; *r* = .61, *p* < .01). In addition, work engagement was positively correlated with career success (*r* = .72, *p* < .01). This shows that in order to improve the career success of male nurses, it is necessary to promote their career identity and strengthen their work engagement, because these variables are closely and positively related.

**TABLE 2:** Correlations among study variables

		1	2	3	4	5	6	7	8	9
1	Work engagement									
2	Career awareness and evaluation	.68**								
3	Career social support	.67**	.88**							
4	Career social skills	.68**	.86**	.87**						
5	Career frustration reaction	.62**	.84**	.85**	.90**					
6	Career self‐reflection	.61**	.79**	.81**	.80**	.82**				
7	Career identity	.70**	.95**	.95**	.95**	.94**	.88**			
8	Career satisfaction	.74**	.77**	.79**	.74**	.71**	.68**	.80**		
9	Career competence	.61**	.72**	.74**	.71**	.70**	.65**	.75**	.72**	
10	Career success	.72**	.80**	.82**	.78**	.76**	.72**	.83**	.92**	.93**

** p < .01.

### 4.3 Verification of research hypotheses

First, we assessed the measurement model which includes three latent constructs (career identity, work engagement and career success). Confirmatory factor analysis revealed that the three‐factor model fit the data well: *χ* ^2^ = 256.99, *df* = 97, *χ* ^2^/*df =* 2.65, CFI = 0.98, TLI = 0.97, RMSEA = 0.05, 90% CI: 0.04–0.06, SRMR = 0.03 (*p* < .01), and all indicators were significantly loaded on the corresponding constructs.

**FIGURE 1:**
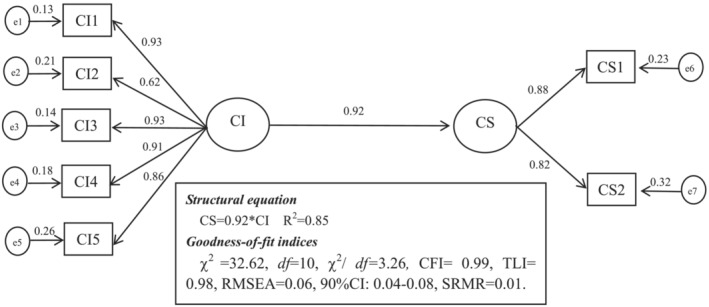
Direct effect model. CI, career identity; e1–e5, manifest variables of the five dimensions of career identity; CS, career success; e6–e7, manifest variables of the five dimensions of career success

Second, we tested a direct effect model to verify whether career identity positively affects career success (Hypothesis 1). The results showed that the direct effect model exhibited a good fit to the data: *χ* ^2^ = 32.62, *df* = 10, *χ* ^2^/*df =* 3.26, CFI = 0.99, TLI = 0.98, RMSEA = 0.06, 90% CI: 0.04–0.08, SRMR = 0.01 (*p* < .01). Figure 1 depicts the direct model. Career identity was found to be positively associated with career success (*β* = .92, *p* < .01). And we performed 2000 bootstrapping resamples to justify the 95% CI of the total direct effect of career identity on career success. The results of bootstrapping showed that the 95% CI for the total direct effect was (0.89, 0.95). Career identity explained 92% of the variance of career success.

**FIGURE 2:**
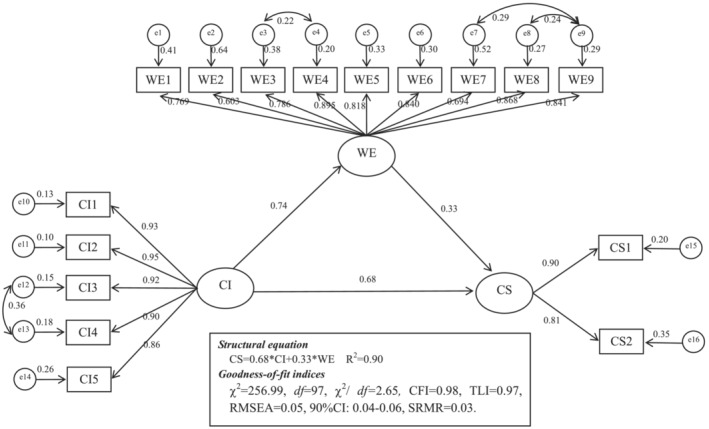
Mediation model. WE, work engagement; CI, career identity; CS, career success

Finally, we tested a mediating effect model to verify whether work engagement mediates the relationship between career identity and career success (Hypothesis 2). We repeated this process 2000 times to arrive at an empirical approximation of the sampling distribution and obtained the estimate and confidence interval for this indirect effect. According to the confirmatory factor analysis, the three‐factor model had an adequate fit to the data with *χ* ^2^ = 256.99, *df* = 97, *χ* ^2^/*df =* 2.65, CFI = 0.98, TLI = 0.97, RMSEA = 0.05, 90% CI: 0.04–0.06, SRMR = 0.03 (*p* < .01), and all indicators were significantly loaded on the corresponding constructs. Figure 2 depicts the mediating effect model. Career identity was positively related to work engagement (*β* = .74, *p* < .01), which in turn had a positive effect on career success (*β* = .33, *p* < .01). Moreover, even after incorporating work engagement, career identity remained significantly related to career success (*β* = .68, *p* < .01), indicating that work engagement played a partial mediating role. We performed 2000 bootstrapping resamples to justify the 95% CI of the indirect effect of career identity on career success via work engagement. The results of bootstrapping showed that the 95% CI of the mediating effect was (0.18, 0.31). Career identity and work engagement together explained 90% of the variance of career success. The indirect effect accounted for 27% of the total effect of career identity on career success (total effect = 0.92, indirect effect = 0.24) (Table 3).

**TABLE 3:** Confidence interval of mediating effect value in chain mediated model (2000 bootstrap samples)

Model path	Estimate	95% CI	
		LLCI	ULCI
CI → CS	0.92	0.89	0.95
CI → WE → CS	0.24	0.18	0.31

It indicates that male nurses' career identity can well predict their career success. At the same time, a better career identity can promote their career success by increasing work engagement. Job engagement plays a good intermediary role between male nurses' career identity and career success. Therefore, nursing managers should not only pay attention to male nurses' career identity but also pay attention to their engagement in the clinical nursing work.

## 5 DISCUSSION

The Chinese male nurses' career success in our study is similar to previous studies which found the level of career success of nurses is low (Dan et al., 2018; Wu et al., 2020; L. G. Zhang & Jin, 2018), and among the male nurses' career success, the score of the career competence dimension is the lowest. The reasons may be that hospital managers do not pay enough attention to the vocational education of male nurses, so male nurses are not clear about their career development planning and direction, and lack awareness and motivation to improve their career competence (Ma et al., 2020; Mao et al., 2020). At the same time, influenced by Chinese social concepts, nurses' social status is low, especially male nurses, which greatly affects their enthusiasm for nursing work and is not conducive to achieving career success (Feng et al., 2017). Although the society emphasizes gender equality, the traditional Chinese concept believes that nursing work is usually undertaken by women. Moreover, in China, the social status and income of nurses are not high, and men often assume the pillar role. If they engage in nursing work, this is contrary to this role. In addition, the male nurse is a minority of the nurse population in the world especially in China, which implies negative influences on their nursing identity and career success because the number of male nurses is small. Because of not given enough attention, they are in the danger of occupational marginalization. This will further exacerbate their low career identity and resignation, forming a vicious circle (Sedgwick & Kellett, 2015).

As the analysis of data in this survey shows, male nurses' career identity can predict their career success very well. To a certain degree, we can conclude that male nurses with good career identity can usually achieve career success. That is because improved career identity can benefit the enthusiasm in nursing work. They will commit themselves to the nursing career more proactively and deliver better service to the patients (Lin et al., 2021; Min et al., 2021). On the other hand, the positive feedback from patients can enhance nurses' sense of responsibility and accomplishment and foster a new career identity (Byram et al., 2021). It forms a virtuous circle. So improving their career identity makes great sense in promoting career success. However, due to the impact of the social concept that the nursing industry is often engaged by women, management of male nurses in hospital is not often underlined (Feng et al., 2019; Zhou et al., 2021). This will affect male nurses' career attitude badly. So managers should assist male nurses to carry out career development planning and life planning education. First, they should be guided to understand themselves and career correctly, enhance career identity and integrate self‐development and nursing career development. It is beneficial to investigating the ideological trend while male nursing students choose this major and taking measures to stabilize their ideology during higher vocational nursing education so as to obtain jobs smoothly (Powers et al., 2018). Meanwhile, the managers can provide some targeted training to male nurses to improve career identity (Arif & Khokhar, 2017).

In this survey, we also find that work engagement mediates the relationship between career identity and male nurses' career success. With a good career identity, the individual is more willing to put time and energy into work and easier to get positive feedback from work and a sense of career success. Sun et al. (2022) found that in clinical nursing work, nurses with high career identity for nursing have high work enthusiasm and engagement. This is consistent with the results of Hirschi's research (Hirschi, 2012). When individuals invest more in their work, they will find a sense of existence and achievement, and that will help to achieve career success (Q. Chen et al., 2021). Therefore, work engagement plays a good intermediary role between male nurses' career identity and career success. So we should not only pay attention to their career identity but also focus on their work engagement level. The nursing manager should minimize the impact of traditional concept, implement the gender equality and improve the compensation and welfare distribution mechanism to let male nurses get well‐matched pay for their work. Managers should also provide enough room for promotion and development to make them truly feel the glory and value of nursing career (Alvares et al., 2020). Meanwhile, work commitment and enthusiasm of male nurses should be sparked by further development of incentive mechanism. Based on their own features and need of department, skill and research training also make sense to improve their nursing competence, personalize and diversify their development. Given full play to their advantages, the male nurses will get higher sense of professional achievements and perform better in their work (M. L. Cheng et al., 2018).

Male nurses can play a better complementary role in the nursing team with female as the main body. They are not only the leading role in emergency room, ICU and so on but also the backbone player in the relief efforts of earthquake, snow disaster and infectious disease (Appiah et al., 2021; Banakhar et al., 2021). Their career identity and work engagement are critical to the quality of health care services. Today, our nursing team is still short of hands (Papantoniou, 2021). Policymakers and managers need to think about how to attract and retain male nurses, provide personalized career path for them and promote their career success. As a result, it is necessary to understand the career identity and work engagement of male nurses to provide inspiration and reference for managers to reduce the loss of male nurses and promote the nursing team development.

## 6 IMPLICATIONS FOR NURSING MANAGEMENT

Our research provides relevant implications for nursing management. After studying the career success of male nurses, which is a special group, we find out that their career identity is positively correlative to career success. Meanwhile, the work engagement plays a mediating role between them. It suggests that nursing managers should pay attention to not only their career identity but also their work engagement. Nursing managers should minimize the impact of traditional concept, implement the gender equality and give moderate care for them. The managers should also improve the management system of male nurses and enhance their work identity to realize the establishment of more professional nursing team of male nurses, gender balance and professional development of nursing. Based on their own features and need of department, skill and research training are beneficial to improving their nursing competence, personalizing and diversifying their development. Given full play to their advantages, the male nurses will perform better in their work. Last but not least, the further exploration of better incentive mechanism also makes sense in promoting male nurses' career identity, work engagement and career success by the reform of performance appraisal mechanism and salary adjustment according to their ability.

## 7 LIMITATION

There are some limitations in our study which need to be improved through follow‐up research. First, our study is conducted in the form of self‐report questionnaire, and the results are relatively subjective. Second, our research is only carried out in part of provinces in China. Therefore, the sample has certain limitations. In the next research, we will further expand the sample size and involved regions, so as to make the sample more representative.

## CONFLICT OF INTEREST

None.

## ETHICAL APPROVAL

This study was conducted under ethical guidelines described in the Helsinki Declaration. The ethics approval was not required because there was no unethical behaviour existed in the study and our study did not involve human clinical trials or animal experiments. Before the investigation, we explained the purpose to the participants, asked for their verbal consent before conducting the survey and signed the informed consent form with them. During the investigation, participants could terminate and withdraw from the investigation at any time and the questionnaire was completed anonymously.

## Data Availability

The data that support the findings of this study are available on request from the corresponding author. The data are not publicly available due to privacy or ethical restrictions.
